# The month of walking alone and BDNF level differ between drug-naive first-episode schizophrenia patients and healthy controls

**DOI:** 10.3389/fnmol.2023.1177524

**Published:** 2023-05-10

**Authors:** Zhenmei Zhang, Lejia Fan, Liu Yuan, Zongchang Li, Lijun Ouyang, Xiaoqian Ma, Zihao Yang, Jingyan Lv, Shuting Chen, Xiaogang Chen, Ying He

**Affiliations:** ^1^National Clinical Research Center for Mental Disorders, Department of Psychiatry, The Second Xiangya Hospital of Central South University, Changsha, Hunan, China; ^2^Hunan Key Laboratory of Psychiatry and Mental Health, China National Technology Institute on Mental Disorders, Institute of Mental Health, Hunan Medical Center for Mental Health, The Second Xiangya Hospital of Central South University, Changsha, Hunan, China

**Keywords:** the month of walking alone, BDNF, neurodevelopment, neurocognitive function, first-episode schizophrenia patients (FEP)

## Abstract

**Introduction:**

Schizophrenia is a neurodevelopmental disorder, characterized by impairment in reasoning, affectivity, and social relationships. Previous studies have shown delayed motor development and Brain-Derived Neurotrophic Factor (BDNF) level change in individuals with schizophrenia. We researched the month of walking alone (MWA) and BDNF level between drug-naive first-episode schizophrenia patients (FEP) and healthy control (HC), as well as how they behave in neurocognitive function and severity of symptoms. Predictors of schizophrenia were further explored too.

**Methods:**

We researched the MWA and BDNF levels between FEP and HCs in the Second Xiangya Hospital of Central South University from August 2017 to January 2020, as well as how they behave in neurocognitive function and the severity of symptoms. A binary logistic regression analysis was used to examine the risk factors affecting the onset and treatment outcome of schizophrenia.

**Results:**

We find that FEP showed a walking delay and lower BDNF levels compared to HCs, which were associated with cognitive impairment and severity of symptoms. According to the difference and correlation analysis results, and combined with the appropriate application conditions for binary logistic regression, Wechsler Intelligence Scale Picture completion, Hopkins Verbal Learning Test-Revised, and Trail Making Test: part A were added to the binary logistic regression analysis to distinguish FEP and HCs.

**Conclusion:**

Our study has shown delayed motor development and changes in BDNF levels in schizophrenia, extending insight into the early identification of patients with schizophrenia versus healthy populations.

## 1. Introduction

Schizophrenia is an etiologically heterogeneous syndrome caused by genetic and environmental factors. It is a psychiatric syndrome characterized by psychotic symptoms of hallucinations, delusions, and disorganized speech, with negative symptoms such as decreased motivation and diminished expressiveness, and cognitive deficits involving impaired executive functions, memory, and mental processing. Schizophrenia affects nearly 1% of the world population and is one of the top 10 global causes of disability (Marder and Cannon, 2019). In the absence of any biological markers, the current diagnosis and treatment of schizophrenia are mainly based on clinical questionnaires (Tomasik et al., 2016), limiting the intended ideal therapeutic effect (Winship et al., 2019).

Although the pathogenesis of schizophrenia remains unclear, the brain’s neurological impairment may play a role. There are some intersections in the cognitive and motor control aspects of the brain. Some brain regions, such as the cerebellum and dorsolateral prefrontal cortex, not only support locomotion (Niendam et al., 2012) but also cognitive control (Diamond, 2000). Infant exercise development is critical to adaptation functions and predictive cognitive results and neurological disorders (Marrus et al., 2018). Six milestones are considered to be universal, fundamental to the acquisition of self-sufficient erect locomotion, and simple to test and evaluate. These are sitting without support, hands-and-knees crawling, standing with assistance, walking with assistance, standing alone, and walking alone. Among these, walking alone is the most important and the behavior of walking generally appears between the 8th and 18th months of life and is gradually refined with practice and maturity (Onis, 2006). Individuals suffering from schizophrenia reached most developmental milestones (e.g., smiling, lifting head, sitting, crawling, and walking) later than the controls and individuals with other psychiatric disorders (Sorensen et al., 2010) while childhood-onset Schizophrenia patients have shown neurodevelopmental barriers as early as 2 years old (Walker et al., 1994). Poor premorbid functioning, including cognitive impairment, social deficit, and movement irregularities, is found in many schizophrenia patients (Schenkel and Silverstein, 2004). Additionally, there is a more serious primary or true motion abnormality in schizophrenia patients than in their unaffected first-degree relatives and healthy controls (Hirjak et al., 2018). Children with definite motor problems were more likely to have had psychotic experiences than children with no definite motor problems (Burton et al., 2023). All of the above information supports the neurodevelopmental hypothesis, which theorizes that the neuropathological process involved in schizophrenia may originate from congenital central nervous system damage and the accumulation of brain developmental defects early in life. This theory is becoming an important etiological basis for schizophrenia (Weinberger, 1987).

In addition to neural circuit, Brain-Derived Neurotrophic Factor (BDNF) is a major contributory factor in regards to neurodevelopment. BDNF plays an important role in the regulation of neurological development while lower BDNF levels were observed in FEP than those of healthy controls (HC) in many studies (Man et al., 2018; Singh et al., 2020). The abnormalities of the BDNF signal may lead to defects in brain function, thereby making individuals more susceptible to schizophrenia. Reduced BDNF levels may have significant implications for neurodevelopmental abnormalities before the emergence of early functional deficits at the onset of psychosis (Singh et al., 2020). BDNF is a critical modulator in the neurodevelopment and maintenance of both the central and the peripheral nervous systems (Jiang et al., 2019). On the other hand, exercise induces BDNF expression and signaling in the hippocampus, promoting learning, and memory formation (El et al., 2019). BDNF level might be associated with the development and function of the motor system, especially in toddlers.

Given these previous findings, we hypothesize that patients with schizophrenia have underlying neurological impairments and motor dysfunctions from childhood, such as reduced BDNF and developmental delay, leaving them with poorer performance in terms of higher-order cognition, social functioning, and treatment outcomes. In the present study, we analyzed the MWA and BDNF levels between the FEP and HC, as well as their neurocognitive behavioral functions and severity of symptoms. Together, our study is expected to provide new perspectives on the early identification of schizophrenia.

## 2. Materials and methods

### 2.1. Participants

This study was conducted in the Second Xiangya hospital of Central South University from August 2017 to January 2020. A total of 100 patients with first-episode schizophrenia and 73 healthy controls were enrolled. The project was approved by the Ethics Committee of the Second Xiangya Hospital of Central South University, and all participants were informed of the content of this study and signed written informed consent.

#### 2.1.1. Study group

First episode schizophrenia Inclusion criteria: ① in accordance with the Diagnostic and Statistical Manual of Mental Disorders-IV-TR, (DSM-IV-TR) of the US Mental Disease Diagnosis (DIAGNOSTIC AND STATISTICAL OF Mental Disorders-IV-TR), using DSM-IV-TR for a diagnosis of schizophrenia; ② first episode with the course of disease < 1 year, have not been treated with antipsychotic medication; ③ age 13–48 years old; and ④ Han nationality, right handedness. Exclusion criteria were the following: ① received anti-psychiatric drugs or electroconvulsive therapy; ② accompanied by other serious psychiatric diseases other than schizophrenia or suffering from serious somatic diseases or organic brain diseases; ③ color blind; ④ in lactation or pregnancy; and ⑤ substance or alcohol abuse/dependence.

#### 2.1.2. Control group

Healthy controls (HC) were recruited from March 2015 to August 2019. Inclusion criteria consist of: ① controls were assessed using the clinical definitive interview non-patient version (SCIDNP), and all controls did not have any history of mental illness; ② no first-degree relatives with mental illness; and ③ Han nationality, right handedness. The exclusion criteria for the control group were the same as that of the study group.

### 2.2. Methods

#### 2.2.1. Tool and observation indexes

##### 2.2.1.1. Survey instrument

Face-to-face completion of a general information questionnaire using a self-administered questionnaire, including gender, age, marital status, years of education, height, weight, birth length, birth weight, maternal pregnancy status (any premature birth, obstructed labor, hypoxia, prolonged labor, maternal alcohol consumption during pregnancy, viral infections and drug use, etc.), any medical history (traumatic brain injury, coma for more than 5 min, convulsions, etc.), medication use, and family history of psychiatric disorders, etc.

##### 2.2.1.2. Cognitive tasks

Wechsler Intelligence Scale (WAIS.I, WAIS.PC), Continuous Performance Test (CPT), part of the Matrics Consensus Cognitive Battery (MCCB), including Trail Making Test: Part A/B (TMT-A/B) were utilized for testing speed of processing. The Hopkins Verbal Learning Test-Revised (HVLT-R) for testing verbal learning and the Continuous Performance Test (CPT) for attention/vigilance were administered to both groups. In order to determine the symptom dimension, Positive and Negative Schizophrenia Symptom Scale (PANSS) assessments including Positive subscale, Negative subscale and General psychopathology subscale, Personal and Social Functioning Scale (PSP), and Gross Assessment of Functioning Scale (GAF) were only administered to the study group. All the assessments (clinical, cognitive, and social performance) were made at the same visit.

#### 2.2.2. Plasma BDNF level analysis

Fasting venous blood was collected from the enrolled subjects in the morning of the day following questionnaire and assessments, and centrifuged at 3,000 rpm for 10 min. The upper layer of plasma was then aspirated into plastic centrifuge tubes, numbered and recorded, and immediately transferred to −80°C refrigerator for freezing and storage for plasma BDNF detection by Enzyme-linked Immunosorbent Assay (ELISA).

#### 2.2.3. Statistical analysis

Statistical Product and Service Solutions (SPSS) 25.0 software was used to perform the statistical analyses. Continuous variables are presented as mean ± SD. Independent samples *t*-test was used for comparison between groups; when the variance was not consistent with homogeneity of variance, the corrected *t*-test (t’ test) was used; the count data was tested by χ2 test. Spearman’s correlation analysis was performed to explore the correlation between the study group’s MWA, BDNF level, clinical information, cognitive function, and symptom severity. A binary logistic regression analysis was used to examine the risk factors affecting the onset and treatment outcome of schizophrenia. All tests were 2-tailed and *p* < 0.05 was considered to have a statistically significant difference. All *p*-values were corrected with the false discovery rate.

## 3. Results

### 3.1. Demographic and clinical characteristics of drug-naive first-episode schizophrenic patients and healthy controls

A total of 100 patients with first-episode schizophrenia were enrolled. The age of The FEP ranged from 13 to 48 years (mean age, 21.91 years; *SD*, 0.65). The average PANSS total scores for study patients on admission were 88.98 (*SD*, 2.49) among which PANSS positive scores were 21.49 (*SD*, 0.73), PANSS negative scores were 23.17 (*SD*, 0.88) and PANSS general pathology scores were 42.13 (*SD*, 0.98). A total of 73 healthy controls between the ages of 13 to 30 (mean age, 20.92 years; *SD*, 0.44) were enrolled. There were no significant differences in age, gender, birth weight, birth length, weight, height, Body Mass Index (BMI), or perinatal history between the two groups (*p* > 0.05). The FEP, however, had fewer years of education than the HC (see Table 1).

**TABLE 1:** Demographic and clinical characteristics between schizophrenia patient group and normal control group.

	HC (*n* = 73)	FEP (*n* = 100)	*P*
Gender (M/F)	42/31	56/44	>0.05
Birth weight (kg)	3.38 ± 0.97	3.45 ± 0.12	>0.05
Birth length (cm)	54.30 ± 1.87	53.33 ± 3.33	>0.05
Weight (kg)	59.77 ± 3.38	6.13 ± 0.90	>0.05
Height (cm)	166.13 ± 1.83	165.13 ± 0.92	>0.05
BMI (kg/m^2^)	21.48 ± 0.96	20.70 ± 0.34	>0.05
Age (years)	20.92 ± 0.44	21.91 ± 0.65	>0.05
Education (years)	13.93 ± 0.38	11.55 ± 0.33	<0.01
MWA (month)	12.29 ± 0.37	13.44 ± 0.40	0.04
Medical history (yes/no)	72:1	90:9	0.03
Perinatal history (yes/no)	72:1	95:4	>0.05
PANSS total score	–	88.98 ± 2.49	-
PANSS positive subscale	–	21.49 ± 0.73	-
PANSS negative subscale	–	23.17 ± 0.88	-
PANSS general psychopathology subscale	–	42.13 ± 0.98	-
PSP	–	36.82 ± 13.88	-
GAF	–	40.11 ± 15.54	-

FEP, patients with schizophrenia; HC, health controls; M/F, male/female; BMI, body mass index; MWA, the month of walking alone; PANSS, Positive and Negative Syndrome Scale; PSP, Personal and Social Functioning Scale; GAF, Gross Assessment of Functioning Scale.

### 3.2. Cognitive function and BDNF level between drug-naive first-episode schizophrenic patients and healthy controls

The FEP performed more poorly in all conducted cognitive tasks compared to the HC, including the speed of processing, attention/vigilance, working memory, and verbal/visual learning (see Table 2 for details). Additionally, the FEP showed a lower level of plasma BDNF than the HC (*p* < 0.05).

**TABLE 2:** Comparisons of cognitive function measurements and BDNF level between schizophrenic patients and healthy control.

Items	HC	FEP	*F*	Corrected *p*-value
WAIS. I	20.85 ± 0.58	16.22 ± 0.77	-4.806	<0.001
WAIS. PC	15.10 ± 0.35	10.53 ± 0.47	-7.885	<0.001
HVLT. R	28.15 ± 0.46	21.49 ± 0.69	-8.000	<0.001
TMT- A (s)	31.30 ± 1.08	48.37 ± 3.45	4.721	<0.001
TMT- B (s)	78.81 ± 3.75	135.98 ± 7.74	6.649	<0.001
STROOP-dots (s)	15.71 ± 0.59	19.05 ± 0.76	3.468	0.001
STROOP-words (s)	16.59 ± 0.44	22.74 ± 1.05	5.401	<0.001
STROOP-colors (s)	27.57 ± 0.90	36.65 ± 1.55	5.053	<0.001
CPT. vision-underreport	3.62 ± 0.54	26.99 ± 4.88	4.763	<0.001
CPT. vision-average completion time (ms)	838.75 ± 6.71	876.24 ± 12.02	2.725	0.010
CPT. hearing-misstatement	17.84 ± 1.25	23.89 ± 1.22	3.441	0.001
CPT. hearing-underreport	20.49 ± 5.84	40.35 ± 5.08	2.577	0.011
CPT. hearing-correct rate (%)	90.86 ± 1.37	81.63 ± 2.13	-3.646	0.001
BDNF (ng/ml)	1407.82 ± 203.55	1011.90 ± 128.26	-2.092	0.036

FEP, patients with schizophrenia; HC, health controls; WAIS. I/PC, Wechsler Adult Intelligence Scale Information/Picture Completion; HVLT.R, Hopkins verbal learning test-revised; TMT, trail making test; CPT, continuous performance test; BDNF, brain-derived neurotrophic factor.

### 3.3. Correlation analysis between the MWA, BDNF, and cognitive function in the drug-naive first-episode schizophrenia patient group

**FIGURE 1:**
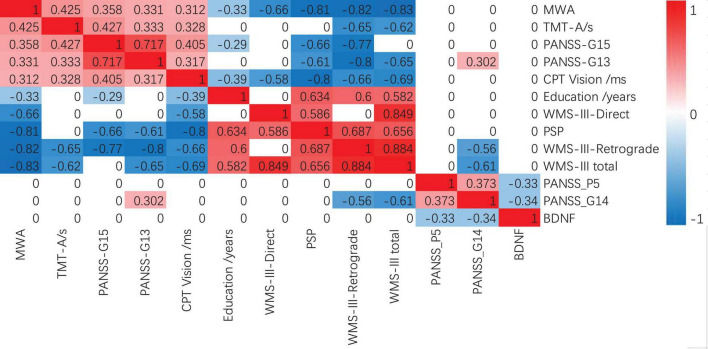
Spearman correlations between MWA, BDNF, and MCCB in FEP. Bonferroni correction *P* < 0.005 s. FEP, patients with schizophrenia; MWA, month of walking alone; BDNF, brain-derived neurotrophic factor; WMS-III, Wechsler Memory Scale-Third Edition; PANSS, positive and negative schizophrenia symptom scale; PSP, Personal and Social Functioning Scale.

As shown in Figure 1, MWA was positively correlated with TMT-A, the average completion time of the CPT Vision test, PANSS-G15 preoccupation and PANSS-G13 disturbance of volition, while negatively correlated with education years, WMS-III, and PSP in FEP. BDNF levels in FEP were negatively correlated with PANSS-P5 exaggeration and PANSS-G14 impulse control impairment.

### 3.4. Multivariable logistic regression

The binary logistic regression equation was used to explore cognitive factors that contributed to schizophrenia. After adjusting the effects of socio-demographic variables including gender, age, and years of education, the binary logistic regression model was fitted to identify the FEP and the HC. Combining the results of the difference and correlation analyses we conducted previously, we selected the following variables for binary logistic regression, which included WAIS. I, WAIS. PC, HVLT. R, TMT- A, TMT- B, STROOP-dots, STROOP-words, STROOP-colors, CPT. vision-underreport, CPT. vision-average completion time, CPT. hearing-misstatement, CPT. hearing-underreport, CPT. hearing-correct rate, BDNF, PANSS-P5 and PANSS-G14 for the binary logistic regression analysis. However, only WAIS. PC (*OR*, 0.819; 95% *CI*, 0.688–0.975; *p* < 0.05), HVLT. R (*OR*, 0.844; 95% *CI*, 0.754–0.943; *p* < 0.05), TMT-A (*OR*, 1.051; 95% *CI*, 1.011–1.091; *p* < 0.05) and the constants (*OR*, 115.139; *p* < 0.05) were significant predictors of the FEP (Table 3).

**TABLE 3:** Significant variables discriminating FEP from HC.

Items	*B*	SE	Wald	Sig	OR	95% CI
WAIS. PC	-0.199	0.089	5.017	0.025	0.819	0.688–0.975
HVLT. R	-0.170	0.057	8.890	0.003	0.844	0.754–0.943
TMT-A/s	0.049	0.019	6.466	0.011	1.051	1.011–1.091
Constants	4.746	1.643	8.345	0.004	115.139	–

*B*, coefficient value; SE, standard error; Wald, Wald statistics; Sig, *P*-value; 95%; OR, odds ratio; CI, 95% confidence interval; WAIS. PC, Wechsler Adult Intelligence Scale Picture Completion; HVLT, Hopkins verbal learning test; TMT, trail making test.

**FIGURE 2:**
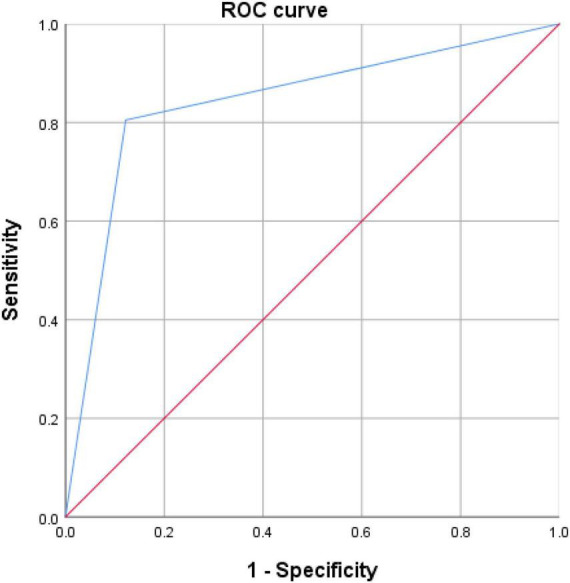
ROC curve of the logistic regression model for predicting the FEP.

The discriminating ability of WAIS.PC, HVLT.R, and TMT-A to separate the FEP from the HC were determined by ROC analysis. Figure 2 represents the ROC curves for utilizing WAIS.PC, HVLT.R, and TMT-A to diagnose schizophrenia. These have a good diagnostic value for predicting the FEP in the whole sample with an AUC of 0.873 (*p* < 0.0001) and a sensitivity and specificity of 72.2 and 93.1%, respectively.

## 4. Discussion

Schizophrenia is a severe mental disorder with varying degrees of cognitive impairments (Mihaljevic-Peles et al., 2019). Our findings were consistent with previous studies that indicated patients with schizophrenia had worse cognitive function than controls in the Chinese population (He et al., 2019; Liu et al., 2019). Our study found that: (1) Compared to the HC, the FEP had fewer years of education, lower IQ, took longer to reach the age of walking without support (months), had more prevalent prior medical cases, and showed worse cognitive functions, including the speed of processing, attention/vigilance, working memory, and verbal/visual learning. (2) The later patients reached the MWA, the poorer they performed on some items of cognitive function and symptom severity. For example, MWA was positively correlated with TMT-A, the average completion time of the CPT Vision test, PANSS-G15, and PANSS-G13, while negatively correlated with education years and PSP in the FEP. Unlike the MWA, the BDNF level was only negatively correlated with PANSS-P5 and G14 in the FEP. (3) According to the difference and correlation analysis results along with the appropriate application conditions of binary logistic regression, WAIS.PC, HVLT.R, and TMT-A were added to the binary logistic regression analysis for differentiating The FEP and HC.

Through these results, we discovered the FEP learned to walk independently at a later age than the HC. In addition, our finding aligns with a previous longitudinal study that suggested delayed attainment of milestones in infancy significantly increased the risk of later development of schizophrenia in a dose-response manner (Clarke et al., 2011). Apart from that, many retrospective studies discovered that adverse premorbid functioning, including cognitive impairment, social deficits, and motor dysphoria, had been identified in the medical histories of many patients with schizophrenia (Schenkel and Silverstein, 2004; Burton et al., 2023). Compared to healthy controls, children with schizophrenia showed stable motor developmental deficits in manual dexterity and a developmental lag in aiming and catching. Children with definite motor problems were more likely to have had psychotic experiences than children with no definite motor problems (Burton et al., 2023). Results of these studies along with our findings indicate that underlying neurodevelopmental defects, as indexed by delayed attainment of milestones, may increase the risk of schizophrenia in adulthood.

Patients who learn to walk later show poorer cognitive function, poorer performance in sustaining continuity and ability to deal with problems, and impaired social function, etc. The MWA reported by parents has been found to significantly predict the subsequent language development speed of children with autism spectrum disorder (Bedford et al., 2016). This suggests that a child’s early motor abilities can have longitudinal and cross-domain effects, which may be related to later cognitive results, such as affecting academic performance and executive function. In terms of PANSS scores, the MWA in the schizophrenia patient group was positively correlated with scores for some general symptoms such as PANSS-G13 (disturbance of volition) and PANSS-G15 (preoccupation). The MWA was negatively correlated with the total score of social functioning. Schizophrenia disturbs many aspects of the life of an individual and brings about deficits in functioning of the cognitive, perceptual, motor and emotional domains, eventually causing social withdrawal in these patients. Adequate social functioning is essential for patients with schizophrenia, as it is for any individual, as it helps them to achieve their life goals (Dziwota et al., 2018). A longitudinal study (He et al., 2014) showed that the increase in PSP score was correlated with the decrease in PANSS general score and PANSS total score. Additionally, exercise had been shown to influence PANSS general score and PANSS total score (Curcic et al., 2017). These were consistent with our expectations that motor function of patients with schizophrenia was associated with both PANSS general score and social function. Thus, we speculated whether there was a possibility that the older the patient learned to walk, the poorer the neurodevelopment process is and the severer the symptoms in these patients. The delay in MWA reflects, to some extent, the low social functioning of the patient. Knowledge of specific contributors to changes in patient functioning may also help to better target symptoms to optimize treatment outcomes.

On the influence of BDNF levels on the cognition and pathogenesis of schizophrenia, BDNF levels in the FEP were negatively correlated with PANSS-P5 exaggeration and PANSS-G14 impulse control impairment as shown in Figure 1. These indicate the FEP with high BDNF levels has lower scores on these two symptom dimensions (exaggeration and impulse control impairment). However, in our results, the correlation between BDNF and cognitive level was not significant. Previous studies also have shown that the mean plasma BDNF level was lower in the FEP than in the HC while BDNF levels in the FEP were negatively correlated with the severity of psychotic symptoms (Heitz et al., 2019; Singh et al., 2020). FEPs had poorer cognitive functions and lower BDNF levels compared to that of controls. Lower BDNF levels were correlated with delayed memory in FEPs compared to high BDNF levels. In remission stages, baseline BDNF levels showed significant correlations with both positive and negative symptoms (Wu et al., 2020). Although changes in serum BDNF levels were related to the improvement in depressive symptoms (Han et al., 2021), baseline BDNF levels were not associated with an improvement in depressive symptoms in patients with schizophrenia (Penadés et al., 2018). Theoretically, the high BDNF level may play a protective factor for schizophrenia and might be associated with the development and function of the motor system, especially in toddlers. In addition to being associated with the development and function of the motor system, BDNF is also a critical modulator in the neurodevelopment and maintenance of both central and peripheral nervous systems (Jiang et al., 2019). On the other hand, exercise induces BDNF expression and signaling in the hippocampus, promoting learning and memory formation (El et al., 2019). Hence, we speculate that the FEP may have developed neurological damage at an earlier age, which might not be prominent in early life and manifests only as neuromotor deficits, such as delayed walking. This will though have a long-term effect on the nervous system, such as decreased BDNF levels, potentially affecting patients’ symptoms and cognitive functions. However, our study did not prove the correlation between plasma BDNF level and the MWA. One possibility is that the sample size is too small to find any related results. Another possible explanation is due to the large time gap as BDNF acts in the critical period of neurodevelopment, especially before 2 years of age, leading to early BDNF level differences, which could change with age. Many factors may contribute to differences in BDNF levels, such as differences in disease status, test material (plasma vs. serum), duration of untreated psychosis, age of onset, duration of disease, physical activity, and differences in the ethnic origins of study subjects (Dinoff et al., 2017). Thus, the role of BDNF levels on cognition and pathogenesis in schizophrenia remains to be investigated further.

In order to distinguish between the two groups of FEP and HC, WAIS.PC, HVLT.R, and TMT-A were utilized along with the binary logistic regression equation. These were conducted in accordance to the difference and correlation analysis results along with the appropriate application conditions of binary logistic regression. ROC analysis was used to measure the discriminatory ability of significant variables for FEP and HC. The ROC curve in Figure 2 shows that WAIS.PC, HVLT.R, and TMT-A have a good diagnostic value for predicting the FEP in the whole sample with an AUC of 0.873. Currently, however, we could not bring the MWA and BDNF into the binary logistic regression, as the MWA or BDNF may not be sufficient to predict schizophrenia accurately. With further clinical data, we could build a more accurate predictive model in the future.

Monitoring early motor developmental milestones and BDNF levels during childhood may still potentially assist in early identification of individuals at risk for psychiatric disorders. Future studies should, however, consider collecting additional indicators related to neurodevelopment. Longitudinal studies are necessary for schizophrenia, and it may be particularly relevant to study neurodevelopmentally related birth cohorts to examine additional early milestones of motor development, such as smiling, head lifting, sitting, crawling, and supporting. The specific timing of walking, unsupported walking, etc. would provide a more detailed and reliable analysis of neurodevelopment. Furthermore, aside from BDNF, other inflammatory factors such as IL-6 and TNF (Boulanger-Bertolus et al., 2018) and the complement system (Sager et al., 2021) are associated with neurodevelopment and schizophrenia, and their correlation analysis with the age of young children needs to be investigated further. The correlation between the age at which young children learn to walk and the level of BDNF and the treatment effect can also be carried out before and after treatment.

### 4.1. Limitations

Some methodological limitations in this study are: ① Our cross-sectional design cannot directly find the causal relationship between the MWA and cognition in the FEP. Cross-sectional survey research cannot collect more detailed and accurate neurodevelopment-related indicators. Retrospective data restricts reliability and is easy to lose, limiting the interpretability of research results. ② Short duration of the study and small sample size resulted in insufficient data and few biochemical blood measurements, especially at BDNF levels. A small sample size limits statistical power and may hinder the detection of differences between groups. BDNF levels were measured after the onset of the patients and did not reflect their effect on neurodevelopment in real-time. ③ We failed to match the years of education between the patient and the control groups. Education levels are significantly correlated with cognitive function. However, considering the poor neurodevelopment and possible difficulties in learning, no correction was made. ④ Future research could be further investigated in imaging studies as well as longitudinal studies involving drug therapy. ⑤ The sample comprises both adults and younger than 18 years old patients (without differentiate both subsamples).

## 5. Conclusion

In summary, cognitive function was lower in the FEP than in HC while the FEP had a walking delay and lower BDNF levels than that of the HC, which were associated with cognitive impairment and symptom severity. WAIS. PC, HVLT. R, TMT-A were added to the binary logistic regression analysis to distinguish between the FEP and HC. We hope that the correlation analysis between BDNF levels and MWA may be statistically significant and that neurodevelopmental indicators such as MWA and BDNF may be used to predict the onset of schizophrenia and treatment outcomes by further expanding the sample and obtaining data on BDNF levels and more accurate data on MWA in early childhood.

## Data availability statement

The data analyzed in this study is subject to the following licenses/restrictions: The data that support the findings of this study are available from the corresponding author upon reasonable request. Requests to access these datasets should be directed to YH, yinghe@csu.edu.cn.

## Ethics statement

The studies involving human participants were reviewed and approved by the Second Xiangya Hospital of Central South University. Written informed consent to participate in this study was provided by the participants’ legal guardian/next of kin.

## Author contributions

All authors were involved in the preparation of the manuscript and approved the final manuscript for publication.
